# Impact of the *Staphylococcus epidermidis *LytSR two-component regulatory system on murein hydrolase activity, pyruvate utilization and global transcriptional profile

**DOI:** 10.1186/1471-2180-10-287

**Published:** 2010-11-12

**Authors:** Tao Zhu, Qiang Lou, Yang Wu, Jian Hu, Fangyou Yu, Di Qu

**Affiliations:** 1 Key laboratory of Medical Molecular Virology of Ministries of Education and Health, Institute of Medical Microbiology and Institutes of Biomedical Sciences, Shanghai Medical College of Fudan University, Shanghai 200032, PR China

## Abstract

**Background:**

*Staphylococcus epidermidis *has emerged as one of the most important nosocomial pathogens, mainly because of its ability to colonize implanted biomaterials by forming a biofilm. Extensive studies are focused on the molecular mechanisms involved in biofilm formation. The LytSR two-component regulatory system regulates autolysis and biofilm formation in *Staphylococcus aureus*. However, the role of LytSR played in *S. epidermidis *remained unknown.

**Results:**

In the present study, we demonstrated that *lytSR *knock-out in *S. epidermidis *did not alter susceptibility to Triton X-100 induced autolysis. Quantitative murein hydrolase assay indicated that disruption of *lytSR *in *S. epidermidis *resulted in decreased activities of extracellular murein hydrolases, although zymogram showed no apparent differences in murein hydrolase patterns between *S. epidermidis *strain 1457 and its *lytSR *mutant. Compared to the wild-type counterpart, 1457*ΔlytSR* produced slightly more biofilm, with significantly decreased dead cells inside. Microarray analysis showed that *lytSR *mutation affected the transcription of 164 genes (123 genes were upregulated and 41 genes were downregulated). Specifically, genes encoding proteins responsible for protein synthesis, energy metabolism were downregulated, while genes involved in amino acid and nucleotide biosynthesis, amino acid transporters were upregulated. Impaired ability to utilize pyruvate and reduced activity of arginine deiminase was observed in 1457*ΔlytSR*, which is consistent with the microarray data.

**Conclusions:**

The preliminary results suggest that in *S. epidermidis *LytSR two-component system regulates extracellular murein hydrolase activity, bacterial cell death and pyruvate utilization. Based on the microarray data, it appears that *lytSR *inactivation induces a stringent response. In addition, LytSR may indirectly enhance biofilm formation by altering the metabolic status of the bacteria.

## Background

*Staphylococcus epidermidis *is an opportunistic pathogen which normally inhabits human skin and mucous membranes, primarily infecting immunocompromised individuals or those with implanted biomaterials. The pathogenicity of *S. epidermidis *is mostly due to its ability to form a thick, multilayered biofilm on polymeric surfaces [1-3]. Treatment of *S. epidermidis *infection has become a troublesome problem as biofilm-associated bacteria exhibit enhanced resistance to antibiotics and to components of the innate host defences [4,5]. Among the *Staphylococci*, the other major human pathogen is *Staphylococcus aureus*, which causes infections ranging from cutaneous infections and food poisoning to life-threatening septicaemia. Aside from biofilm, *S*. *aureus *produce a large array of exotoxins and exoezymes [6].

Two-component regulatory systems (TCSs) play a pivotal role in bacterial adaptation, survival, and virulence by sensing changes in the external environment and modulating gene expression in response to a variety of stimuli [7-9]. Among the TCSs identified in the genomes of *S. epidermidis*, functions of LytSR are unknown, though in *S*. *aureus *LytSR has been demonstrated to play a role in bacterial autolysis and biofilm formation.

LytSR two-component regulatory system was firstly identified from the *S*. *aureus *genome. The *lytS *integration mutant of *S*. *aureus *strain NCTC 8325-4 exhibited a marked propensity to form aggregates in liquid culture and an increased rate of penicillin-and Triton X-100-induced lysis. In combination with subsequent zymographic analysis, it was suggested that LytSR is involved in either regulation of murein hydrolases gene expression or modulation of murein hydrolase activity [10]. Recently, Shrama et al. reported that a *lytS *knockout mutant of *S*. *aureus *strain UAMS-1 produced more adherent biofilm [11].

In search of genes regulated by LytSR in *S*. *aureus*, two additional open reading frames immediately downstream from *lytS *and *lytR *were identified and designated gene *lrgA *and *lrgB*, whose transcription was positively regulated by LytSR and the global regulators Agr and SarA. It was proposed that LrgA, and possibly LrgB, functions in a similar way to an antiholin, i.e., blocking murein hydrolases access to the substrate peptidoglycan [12]. Bayles et al. put forward the possibility that LrgAB exploits a molecular strategy, which is functionally analogous to that mediated by the eukaryotic Bcl-2 family of apoptosis regulatory proteins, to control bacterial programmed cell death [13,14]. Recent study suggested that LytSR regulatory system sense a collapse in membrane potential and then induce the transcription of the *lrgAB *operon [15].

Several TCSs of *S. aureus*, such as *agr *and *arlRS*, have been proven to affect biofilm formation, whereas little has been known in the case of *S. epidermidis*. In *S. aureus and S. epidermidis*, an *agr *mutant forms a significantly thicker biofilm. However, the *agr *regulons of the two species comprise different genes. Autolysin E (AtlE) which has been documented to mediate initial attachment of *S. epidermidis *to a polymer surface, overexpresses in an *agr *mutant, whereas the homologus Atl protein in *S. aureus *is not under *agr *control [16,17]. Previous studies have shown that *arlS *mutation in *S. aureus *enhanced biofilm formation on a polystyrene surface in a complex TSB medium [18]. However, an *arlS *knockout mutant of *S. epidermidis *generated by our laboratory displayed significantly reduced ability of biofilm formation [19], which suggest *S. aureus *and *S. epidermidis *adopt different strategies to regulate biofilm formation even though the genome of *S. epidermidis *is highly homologous to that of *S. aureus *[6].

Therefore, to investigate the role of LytSR in bacterial autolysis and biofilm development in *S. epidermidis*, 1457*ΔlytSR*strain was constructed. The transcriptional profile of 1457*ΔlytSR* was subsequently analyzed by DNA microarray and related functions were examined.

## Results

### Construction of *S. epidermidis *1457*ΔlytSR* and the complementation strain

**Figure 1:**
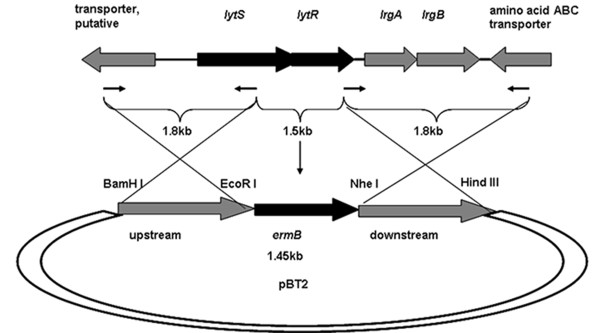
**Physical map of the *lytSR *operon of *S. epidermidis 1457 *and construction of *lytSR *knockout mutant**. Arrows depict open reading frames and indicate their orientations. *lytSR *operon were replaced with the erythromycin resistance gene (*ermB*) as indicated. The *ermB *gene and chromosomal regions flanking the corresponding deletions were amplified by PCR and cloned into plasmid pBT2, yielding the integration vectors pBT2-*ΔlytSR*. The crosses indicate the sites of homologous recombination.

Because *lytSR *has been identified as a regulator of autolysis in *S. aureus*, we hypothesized that *lytSR *control the rate of autolysis in *S. epidermidis*, and may be related with biofilm formation. To test the possibility, *lytSR *knock-out strategy was applied. *S. epidermidis *1457 was used in the present study. We firstly analyzed *lytSR *operon in *S. epidermidis *stains RP62A, ATCC12228, and 1457. The *lytSR *operon was amplified from *S. epidermidis *1457 by PCR with the primers designed according to the *S. epidermidis *RP62A genome sequence, and shares more than 99% nucleotide identity with that in *S. epidermidis *strains RP62A and ATCC12228. BLAST searches indicated that the *lytSR *operon is extensively distributed in gram-positive bacteria. Immediately downstream of *lytR *locates the *lrgAB *operon predicted to encode two potential membrane associated proteins that are similar to bacteriophage holin proteins (Figure 1), as found in *S. aureus *[20].

**Figure 2:**
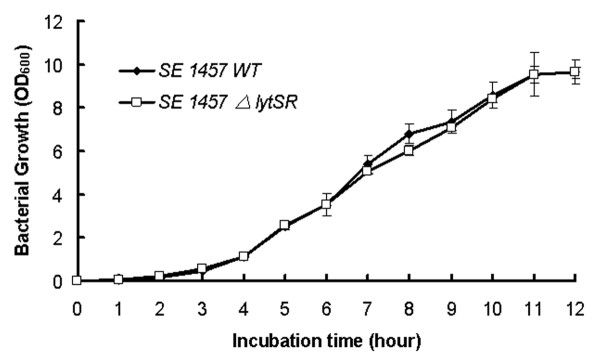
**Growth curves of *S. epidermidis *1457*ΔlytSR***. Bacterial cultures were grown in TSB medium at 37 °C, and growth was monitored by measuring the turbidity of the cultures at 600 nm. Data are means ± SD of 3 independent experiments.

**Figure 3:**
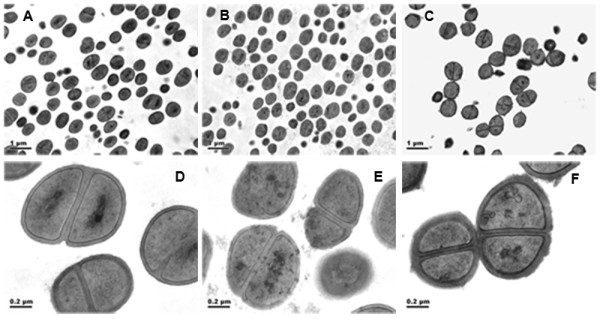
**Morphology of *S. epidermidis *1457*ΔlytSR *under transmission electron microscope**. Strains of *S. epidermidis *1457, *ΔlytSR *and *ΔatlE *were cultured in TSB till stationary phase, fixed with 2.5% glutaraldehyde in Dulbecco's phosphate-buffered saline (PBS). Thin sections were stained with 1% uranyl acetate-lead acetate and observed under a Philips Tecnai-12 Biotwin transmission electron microscope. A-C ×8,200 magnification of 1457, *ΔlytSR *and *ΔatlE *cells respectively; D-F ×43,000 magnification of 1457, *ΔlytSR *and *ΔatlE *cells respectively.

The *lytSR *knockout mutant of *S. epidermidis *1457 was generated by allelic replacement, wherein the *ermB *gene replaced the predicted histidine kinase domain of *lytS *and *lytR *gene (Figure 1). The *lytSR *knockout mutant was then verified by direct PCR sequencing (Additional file 1, Figure S1) and biochemical tests (GPI Vitek card). To rule out an influence of second site mutations on the following findings, the complementation plasmid pNS-*lytSR *was constructed and then electroporated into the mutant, whereas introducing the empty vector pNS as a negative control. Deletion of *lytSR *did not result in a significant growth defect, indicating that *lytSR *is not essential for bacterial cell growth (Figure 2). The morphology of 1457*ΔlytSR* in stationary phase was observed with transmission electron microscope. It revealed that the cell surface was rough and diffused, suggesting alterations in its cell wall surface components (Figure 3). Except for diffused cell surface, the *ΔatlE *strain had a remarkably thickened cell wall (Figure 3).

### Modulation of *lytSR *on murein hydrolase activity

**Figure 4:**
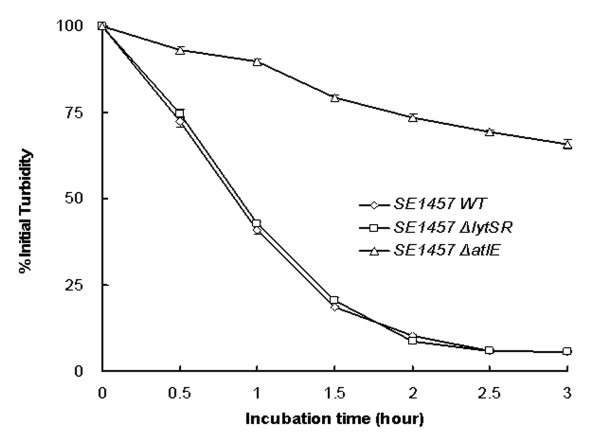
**Autolysis assay of *S. epidermidis *1457*ΔlytSR***. Bacterial cells were collected from early exponentially growing cultures (OD_600 _= 0.7) containing 1 M NaCl, washed twice with ice-cold water and resuspended in an equal volume of Tris-HCl(pH 7.2) containing 0.05%(vol/vol) Triton X-100. The rate of autolysis was measured as the decline in optical density. The *atlE *knockout mutant was used as a negative control. Data are means ± SD of 3 independent experiments.

It has been reported that in *S. aureus lytSR *mutation increased susceptibility to Triton X-100 induced autolysis, therefore, we investigated effect of *lytSR *knockout on autolysis in *S. epidermidis*. Triton X-100 induced autolysis of bacterial cells was carried out, the *atlE *knockout mutant as a negative control. No difference was found between 1457*ΔlytSR* and its parent strain in the Triton X-100 induced autolysis, inconsistent with that observed in *S. aureus *[10], while the negative control *atlE *knockout mutant was resistant to autolysis (Figure 4).

**Figure 5:**
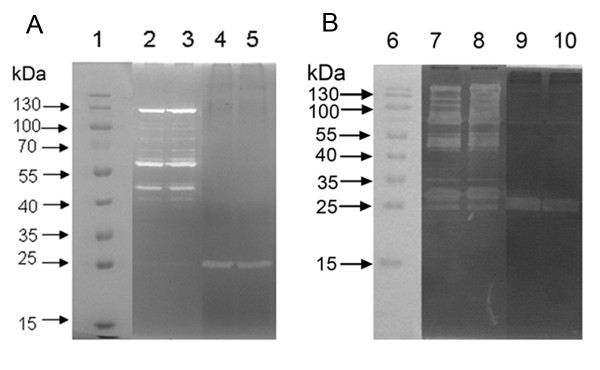
**Zymographic analysis of *S. epidermidis *1457*ΔlytSR***. Extracellular and cell surface proteins were isolated, and 30 μg of each was separated in SDS-polyacrylamide gel electrophoresis gels containing 2.0 mg of *M. luteus *(A) or *S. epidermidis *(B) cells/ml. Murein hydrolase activity was detected by incubation overnight at 37 °C in a buffer containing Triton X-100, followed by staining with methylene blue. Lanes: 1 and 6, molecular mass marker; 2 and 7, cell wall protein from 1457*ΔlytSR *strain; 3 and 8, cell wall protein from wild type strain; 4 and 9, extracellular protein from 1457*ΔlytSR *strain; 5 and 10, extracellular protein from wild type strain. The results are representative of three independent experiments.

Given that the *lytS *mutation in *S. aureus *has pleiotropic effects on different murein hydrolase activity, zymographic analysis using SDS-PAGE incorporated with 2% w/v *M. luteus *(Figure 5A) or *S. epidermidis *(Figure 5B) cells was performed to analyze the activities of extracelluar and cell wall-associated murein hydrolases isolated from bacterial stationary-phase cultures. No significant difference was observed in the zymographic pattern of murein hydrolases between 1457*ΔlytSR* and the parent strain, regardless of *M. luteus *or *S. epidermidis *being taken as the main indicator.

**Figure 6:**
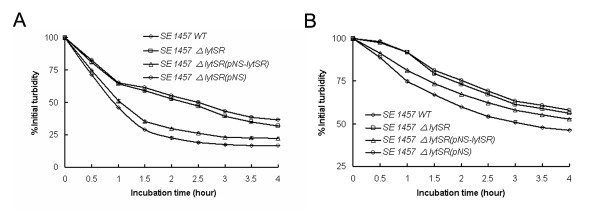
**Quantitative murein hydrolase assays of *S. epidermidis *1457*ΔlytSR***. Aliquots (100 μg) of the extracellular proteins concentrated by ultrafiltration from the supernant were added to a 1-mg/ml suspension of *M. luteus *(A) and *S. epidermidis *(B) cells separately, and the turbidity at 600 nm was monitored for 4 h. Cell wall hydrolysis was determined by measurement of turbidity every 30 min. Data are means ± SD of 3 independent experiments.

Quantitative murein hydrolase assay was further carried out by adding 100 μg of extracellular protein extract to a suspension of heat-killed *M. luteus *or *S. epidermidis *in Tris-HCl buffer, and monitoring the reduction in the suspension turbidity (OD_600_). However, cell wall hydrolysis performed with extracellular murein hydrolases from 1457*ΔlytSR*was undergoing more slowly than that from the parent strain. After 4 hours' incubation, a decrease of 69% or 44% in turbidity (OD_600_) was observed in the suspension of *M. luteus *(Figure 6A) or *S. epidermidis *(Figure 6B) added with extracellular murein hydrolases from 1457*ΔlytSR*, contrasted to a reduction of 84% or 54% with extracellular murein hydrolases from the parent strain, indicating that disruption of *lytSR *resulted in decreased activities of extracellular murein hydrolases (Student's t test, P < 0.05) which probably could not be detected by zymographic analysis. Expression of *lytSR in trans *restored extracellular murein hydrolase activity to nearly wild-type levels (Figure 6).

### Impact of *lytSR *knockout on *S. epidermidis *biofilm formation

**Figure 7:**
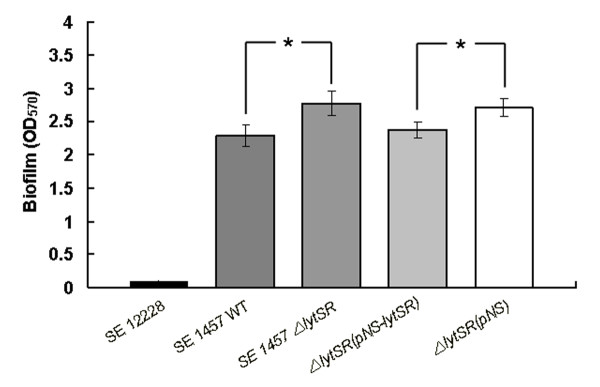
**Effect of *lytSR *gene knocking out on *S. epidermidis *biofilm formation**. The biofilm formation of *S. epidermidis ΔlytSR* and its parent strain was detected by semi-quantitative microtiter plate assay. Briefly, the overnight bacterial were diluted by 1:200 and cultured in 96-well plate (200 μl/well) at 37 °C for 24 h. The well was washed by PBS for 3 times, fixed by 99% methanol and stained with crystal violet. Data are means ± SD of 3 independent experiments. *P < 0.05; *ΔlytSR* vs. WT; *ΔlytSR*(pNS*-lytSR) *vs. *ΔlytSR*(pNS*-lytSR)*.

As biofilm formation is the major determinant of *S.epidermidis *pathogenicity, the impact of *lytSR *deletion on biofilm formation was further investigated. Semi-quantitative assay of *S.epidermidis *biofilm formation in polystyrene microtitre plates was performed and *S.epidermidis *ATCC12228 was used as a biofilm negative control. It was observed that 1457*ΔlytSR* produced slightly more biofilm than the wild-type counterpart (Student's t test, P < 0.05). When *lytSR *was complemented in the mutant, biofilm formation was reduced to the same levels as that observed in the parent strain (Figure 7).

**Figure 8:**
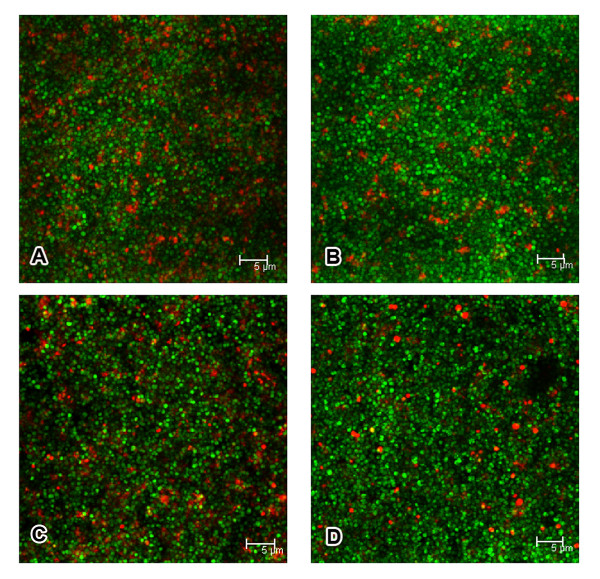
**Confocal photomicrographs of 24-hour-old biofilms**. Biofilms containing *S. epidermidis *1457 strains wild-type (A), *ΔlytSR *(B), *ΔlytSR*(pNS-*lytSR*) (C) and *ΔlytSR*(pNS) (D) were visualized by using the live/dead viability stain (SYTO9/PI). Green fluorescent cells are viable, whereas red fluorescent cells have a compromised cell membrane, as indicative of dead cells. Scale bars = 5 μm. The result is a stack of images at approximately 0.3 μm depth increments and represents one of the three experiments.

**Figure 9:**
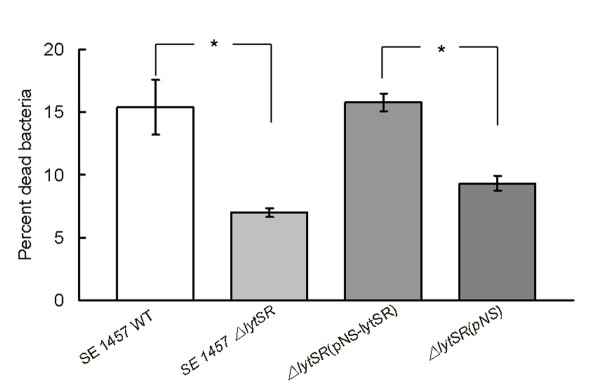
**Quantitative analysis of bacteria cell death in 24-hour-old biofilms**. Live/dead stained biofilm cells were scraped from the dish and dispersed by pipetting. The integrated intensities of the green (535 nm) and red (625 nm) emission of suspensions excited at 485 nm were measured and the green/red fluorescence ratios (Ratio^R/G^) were calculated. The percentage of dead cells inside biofilm was determined by comparison to the standard curve of Ratio^R/G ^versus percentage of dead cells. Data are means ± SEM of 3 independent experiments. *P < 0.05; *ΔlytSR* vs. WT; *ΔlytSR*(pNS*-lytSR) *vs. *ΔlytSR*(pNS*-lytSR)*.

We further examined cell viability inside biofilm of 1457*ΔlytSR* and the wild-type strain by using a fluorescence-based Live*/*Dead staining method. With an appropriate mixture (1:1, m/m) of the SYTO 9 (green) and PI (red), bacteria with intact cell membranes were stained fluorescent green, whereas bacteria with damaged membranes were stained fluorescent red. Significantly decreased level of red fluorescence was observed inside biofilm of 1457*ΔlytSR*, comparing with that inside biofilm of the wild-type strain, as shown in Figure 8. Complementation of 1457*ΔlytSR* with plasmid pNS-*lytSR *restored the level of red fluorescence to that observed inside biofilm of the wild-type strain (Figure 8C, D). A quantitative method based on measuring the red/green fluorescence ratio was carried out to determine the relative cell viability inside biofilm. The percentage of dead cells inside 24-hour-old biofilms of 1457*ΔlytSR* and the wild-type strain were 6% and 15% respectively, as shown in Figure 9. Inside the biofilm of *lytSR *complementation strain, the percentage of dead cells was restored nearly to the wild-type level.

### Transcriptional profiling of 1457*ΔlytSR* strain

To investigate the regulatory role of LytSR, we used custom-made *S. epidermidis *GeneChips to perform a transcriptional profile analysis of the wild type and 1457*ΔlytSR* strains. Two criteria including 2-fold or greater change in expression level and P < 0.05 were employed to select the genes with significantly different expression. It was found that expression of 164 genes was affected by *lytSR *mutation, in which 123 were upregulated and 41 were downregulated. Transcription of *lrgAB *decreased drastically in 1457*ΔlytSR*, indicating that the operon was activated by LytSR in *S.epidermidis*, consistent with the finding for *S. aureus*. Further analysis of the microarray data showed that genes upregulated in the 1457*ΔlytSR* strain included these involved in purine biosynthesis (*pur*; SERP0651-SERP0657), amino acid biosynthesis (*leu*; SERP1668-SERP1671, *hisF, argH, gltB*) and membrane transport (*oppC*, *modC*, *gltS*, *putP*, SERP0284, SERP0340, etc.). Whereas, genes downregulated contained these involved in pyruvate metabolism (mqo-2, SERP2169 and mqo-3), anaerobic growth (*nar*; SERP1985-SERP1987, *arc*; SE0102-SE0106) (Table 1). In addition, genes responsible for encoding ribosomal proteins which make up the ribosomal subunits in conjunction with rRNA were found to be downregulated in 1457*ΔlytSR* (Table 1), consistent with that reported in transcriptional profiling studies of *S. aureus *by Sharma et al. [11]. Transcription of *lrgAB *decreased drastically in 1457*ΔlytSR*, indicating that the operon was activated by LytSR in *S.epidermidis*, consistent with the finding for *S. aureus*. We also noticed that expression of an AraC family transcriptional regulator homologue was remarkably higher in the mutant (Table 1). The microarray experiments were repeated by Prof. Jacques Schrenzel (Genomic Research Laboratory, University of Geneva Hospitals, Switzerland). Transcription of genes required for amino acid biosynthesis, carbon metabolism and membrane transport was also found to be altered in the mutant. Moreover, differential expression of general stress protein, alkaline shock protein 23 and cold shock protein was observed in the latter microarray data. Taken together, it suggested that LytSR may be involved in sensing and responding to changes in the metabolic state of the bacteria.

**Table 1:** Genes expressed differentially in strain 1457*ΔlytSR *compared to the wild-type strain

ORF	Gene name	Description or predicted function	Expression ratio (Mutant/WT)
**Amino acid biosynthesis**			
SERP0034	metE	5-methyltetrahydropteroyltriglutamate homocysteine methyltransferase	2.096
SERP0108	gltB	glutamate synthase large subunit	2.405
SERP0548	argH	argininosuccinate lyase	5.03
SERP1103	aroK	shikimate kinase	2.274
SERP1668	ilvC	ketol-acid reductoisomerase	2.087
SERP1669	leuA	2-isopropylmalate synthase	2.344
SERP1670	leuB	3-isopropylmalate dehydrogenase	2.229
SERP1671	leuC	3-isopropylmalate dehydratase small subunit	11.45
SERP2301	hisF	imidazoleglycerol phosphate synthase, cyclase subunit	5.429
**Amino acid transport**			
SERP0392		di-tripeptide transporter, putative	3.362
SERP0571	oppC	oligopeptide transport system permease protein OppC	12.38
SERP0950		peptide ABC transporter, ATP-binding protein, putative	3.383
SERP1440	putP	proline permease	2.124
SERP1935	gltS	sodium:glutamate symporter	3.267
**Inorganic ion transport and metabolism**			
SERP0284		Na+/H+ antiporter, MnhD component, putative	3.294
SERP0287		Na+/H+ antiporter, MnhG component, putative	2.576
SERP0660		cobalt transport family protein	2.718
SERP1777		iron compound ABC transporter, iron	2.383
SERP1859	modC	molybdenum transport ATP-binding protein	3.294
SERP2428	arsA	arsenical pump-driving ATPase	3.274
**Protein synthesis**			
SERP0721	pheS	Phe-tRNA synthetase alpha chain	2.036
SERP1809	infA	translation initiation factor IF-1	0.5
SERP1812	rplO	ribosomal protein L15	0.482
SERP1813	rpmD	ribosomal protein L30	0.333
SERP1814	rpsE	30 S ribosomal protein S5	0.37
SERP1815	rplR	50 S ribosomal protein L18	0.323
SERP1816	rplF	50 S ribosomal protein L6	0.332
SERP1817	rpsH	30 S ribosomal protein S8	0.357
SERP1818	rpsN-2	30 S ribosomal protein S14	0.306
SERP1819	rplE	50 S ribosomal protein L5	0.324
SERP1821	rplN	50 S ribosomal protein L14	0.346
SERP1820	rplX	50 S ribosomal protein L24	0.356
SERP1822	rpsQ	30 S ribosomal protein S17	0.344
SERP1823	rpmC	50 S ribosomal protein L29	0.332
SERP1824	rplP	50 S ribosomal protein L16	0.438
SERP1825	rpsC	30 S ribosomal protein S3	0.345
SERP1826	rplV	50 S ribosomal protein L22	0.374
SERP1827	rpsS	30 S ribosomal protein S19	0.385
SERP1828	rplB	50 S ribosomal protein L2	0.421
SERP1829	rplW	50 S ribosomal protein L23	0.424
**Nucleotide metabolism**			
SERP0070	guaA	bifunctional GMP synthase/glutamine amidotransferase protein	2.546
SERP0651	purC	phosphoribosylaminoimidazole-succinocarboxamide synthase	2.036
SERP0654	purL	phosphoribosylformylglycinamidine synthetase	2.341
SERP0655	purF	phosphoribosylpyrophosphate amidotransferase	2.164
SERP0656	purM	phosphoribosylformylglycinamidine cyclo-ligase	2.369
SERP0657	purN	IMP cyclohydrolase	2.111
SERP1003	thyA-1	thymidylate synthase	2.014
SERP1810	adk	adenylate kinase	0.444
**Energy metabolism**			
SE0102-12228		carbamate kinase, putative	0.259
SE0104-12228		transcription regulator Crp/Fnr family protein	0.343
SE0106-12228	arcA	arginine deiminase	0.301
SERP0672	cydA	cytochrome d ubiquinol oxidase subunit II-like protein	13.85
SERP1985	narJ	nitrate reductase delta chain	0.441
SERP1986	narH	nitrate reductase beta chain	0.327
SERP1987	narG	nitrate reductase alpha chain	0.324
SERP1990	nirB	nitrite reductase nitrite reductase	0.354
SERP2168	mqo-2	malate:quinone oxidoreductase	0.317
SERP2169		hypothetical protein	0.0165
SERP2261	manA-2	mannose-6-phosphate isomerase	0.479
SERP2312	mqo-3	malate:quinone oxidoreductase	0.451
SERP2352	arcC	putative carbamate kinase	0.427
**DNA replication, recombination and repair**			
SERP0558		ISSep1-like transposase	4.66
SERP0599		site-specific recombinase, resolvase family	2.352
SERP0892		IS1272, transposase	2.774
SERP0909	lexA	SOS regulatory LexA protein	2.227
SERP1023		DNA replication protein DnaD, putative	2.049
SERP2474	hsdR	type I restriction-modification system, R subunit	46.79
**Transcriptional regulator**			
SERP0635		transcriptional regulator, MarR family	3.216
SERP1879		transcriptional regulator, AraC family	21.2

* The entire list of differentially expressed genes can be found on the National Center for Biotechnology Information Gene Expression Omnibus (GEO, available at http://www.ncbi.nlm.nih.gov/geo/ and is accessible through GEO Series accession number GSE20652

The altered expression of five of the genes identified by microarray analysis (*lrgA*, *arcA*, *ebsB*, *leuC*, SERP2169) in 1457*ΔlytSR* were confirmed by real-time RT-PCR with *gyrB*, a housekeeping gene, as the internal control, as shown in Table 2.

**Table 2:** Expression of genes regulated by LytSR confirmed by RT Real-time PCR

Gene	Description	n-fold(microarray)	n-fold(Real time PCR)
*lrgA*	holin-like protein LrgA	0.277	0.133 (0.124, 0.143) ***
SERP2169	hypothetical protein	0.0165	0.013 (0.008, 0.02) ***
*arcA*	arginine deiminase	0.301	0.476 (0.377, 0.601) **
*ebsB*	cell wall enzyme EbsB, putative	0.091	0.278 (0.21, 0.369) **
*leuC*	3-isopropylmalate dehydratase small subunit	11.45	3.85 (3.595, 4.124) **

* Data are means ± SD of 3 independent experiments. ***P < 0.001; **P < 0.01; *ΔytSR1 *vs. WT.

### Pyruvate utilization of 1457 and 1457*ΔlytSR*

**Figure 10:**
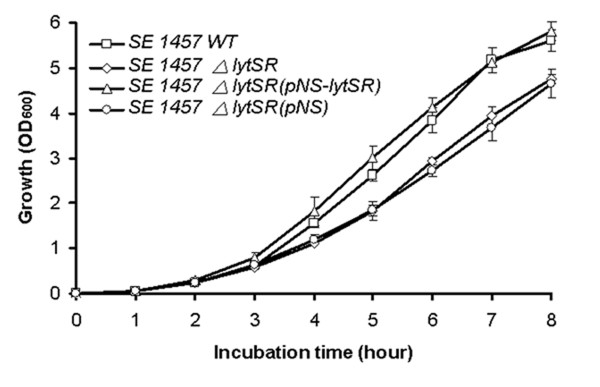
**Pyruvate utilization test of *S. epidermidis *1457*ΔlytSR***. Bacteria were grown in pyruvate fermentation broth at 37 °C, and growth was monitored by measuring the turbidity of the cultures at 600 nm as described in Materials and Methods. Data are means ± SD of 3 independent experiments.

Ability of 1457*ΔlytSR*to utilize pyruvate was found to be impaired by using the Vitek GPI Card system. Meanwhile, expression of genes involved in pyruvate metabolism such as mqo-3, mqo-2 and its neighboring unknown gene SERP2169 were remarkably reduced. For examining the ability to utilize pyruvate, strains 1457 and 1457*ΔlytSR*were cultured in pyruvate fermentation broth and bacterial growth was monitored. The 1457*ΔlytSR* displayed a significantly growth defect in pyruvate fermentation broth, whereas introducing plasmid pNS-*lytSR *into the mutant restored the phenotype, as shown in Figure 10.

## Discussion

The capacity of *Staphylococci *to produce a biofilm is determined by environmental factors, such as glucose, osmolarity, ethanol, temperature and anaerobiosis etc, which suggests that there is a mechanism that senses and responds to extracellular signals [21]. Two-component regulatory systems, composed of histidine kinases and their cognate response regulators, are the predominant means by which bacteria adapt to changes in their environment [7]. Previous studies have shown *yycG/yycF *two-component system is essential for cell viability in *B. subtilis *and *S*. *aureus *and positively controls biofilm formation [22-24]. Another two TCSs of *S. aureus*, *agr *and *arlRS*, have also been proven to regulate biofilm formation [16-18].

Seventeen pairs of TCSs have been determined in the genome of *S. epidermidis *ATCC35984 (RP62A), while 16 pairs in ATCC12228 [25]. We identified one pair of TCS encoding LytS and LytR homologs described in *S. aureus *[10]. The LytSR two-component system in *S. aureus *has been viewed as an important regulator of bacterial autolysis [20]. In the present study, the function of the *S. epidermidis lytSR *opreon was firstly investigated. The *lytSR *knockout mutation did not alter the susceptibility of strain 1457 to Triton X-100-induced lysis, which is different from the finding for *S. aureus *strain NCTC 8325-4 reported by Brunskill et al.[10]. Recently, they found that in the strain UAMS-1, *lytS *knock-out did not result in spontaneous and Triton X-100-induced lysis increasing [11]. The variation in susceptibility to Triton X-100-induced lysis between different staphylococcus strains could be explained partly by the fact that they represent different genetic background.

Since that *lytS *mutation in *S. aureus *has pleiotropic effects on different murein hydrolase activity [20], we hypothesized that in *S. epidermidis*, *lytSR *regulates murein hydrolase activity in a similar manner. Zymographic analysis revealed no significant differences between 1457*ΔlytSR* and the parent strain in the activities or expression of murein hydrolase isolated from both extracellular and cell wall fraction. However, quantification of the extracellular murein hydrolase activity produced by these strains demonstrated that 1457*ΔlytSR* produced diminished overall activity compared to that of the parental strain. As expected, microarray analysis revealed that *lrgAB *opreon was downregulated in 1457*ΔlytSR*. In *S. aureus*, LrgAB has a negative regulatory effect on extracellular murein hydrolase activity and disruption of *lrgAB *led to a significant increase in the activity [10,12]. *cidAB *operon, which encodes the holin-like counterpart of the *lrgAB *operon, and *alsSD *operon, which encodes proteins involved in acetoin production, were then identified. Mutation of either *cidAB or alsSD *operon in the *S*. *aureus *strain UAMS-1 caused a dramatic decrease in extracellular murein hydrolase activity [26,27]. We, therefore, speculate that in *S. epidermidis *some other LytSR regulated proteins similar to CidAB and/or AlsSD, may exist and overcome negative effect imposed by LrgAB on extracellular murein hydrolase activity, which warrants further investigation.

The role of cell death and lysis in bacterial adaptive responses to circumstances has been well elucidated in a number of bacteria, such as *S. aureus and P. aeruginosa*. Webb et al. proposed that in *P. aeruginosa *cell death benefited a subpopulation of surviving cells and therefore facilitated subsequent biofilm differentiation and dispersal [28-30]. Moreover, genomic DNA released following bacterial lysis constitutes the skeleton of biofilm. Since LytSR positively regulates the activity of extracellular murein hydrolases, it may affect cell viability and function in biofilm formation. By using the CLSM, significant decrease in red fluorescence was observed inside biofilm of 1457*ΔlytSR*, which indicated reduced loss of cell viability. Quantitative analysis showed that the percentage of dead cells inside biofilm of the wild type strain was approximately two times higher than that in the mutant. The results are consistent with the observation that 1457*ΔlytSR* displayed a reduction in activity of extracellular murein hydrolases. Disruption of either *cidA *or *alsSD *genes on the *S. aureus *chromosome resulted in significantly decreased extracellular murein hydrolase activity compared with that of the parental strain, UAMS-1. Both the *cidA *and the *alsSD *mutant displayed reduced cell death in stationary phase and completely abrogated cell lysis relative to UAMS-1 [26,27]. Along these lines, the present study confirmed a connection between extracellular murein hydrolase activity and bacterial cell death. Furthermore, expression of *cidC *gene encoding pyruvate oxidase was found to be downregulated (5.07 fold) in 1457*ΔlytSR* through the microarray analysis. Deletion of *cidC *in *S. aureus *or *S. pneumoniae *caused reduced cell death and lysis in stationary phase[31,32]. Based on these data, it was suggested LytSR may play an important role in bacterial cell death and lysis inside biofilm.

In this study, 1457*ΔlytSR*was found to have growth defect in pyruvate fermentation broth and introducing plasmid encoding LytSR (pNS-*lytSR) *into the mutant completely restored the phenotype. Based on the fact that the wild-type strain and the mutant grow equally well in TSB containing 0.25% glucose. As we know, glucose is catabolized by glycolysis to pyruvate. If 1457*ΔlytSR*is impaired in its ability to metabolize pyruvate, then this would be reflected in the growth curve in TSB medium. The data actually indicated that 1457*ΔlytSR*is impaired in the transport of pyruvate and probably amino acids. Previous studies regarding bacterial cells taking up carboxylic acid from the surrounding medium have shown that pyruvate is actively transported across the bacterial membrane and that proton motive force (PMF) plays an important role in the process [33]. In addition, transcription of genes involved in pyruvate metabolism such as mqo-3, mqo-2 and its neighbouring unknown gene SERP2169 were significantly downregulated in 1457*ΔlytSR*. These data along with the findings that in *S. aureus *LytSR responds to a collapse in Δψ by inducing the transcription of the *lrgAB *operon led us to hypothesize that LytSR accelerates pyruvate transport by sensing a reduction in PMF.

Compared to the parent stain, 1457*ΔlytSR*exhibited decreased expression of ribosomal genes and increased expression of amino acid biosynthetic genes, amino acyl-tRNA synthase genes, and amino acid transporters genes, which implies that *lytSR *mutation may induce a stringent response. Additionally, transcriptional profiling studies performed in Switzerland revealed that expression level of genes involved in stress response and cold shock was altered in the mutant. When bacteria encounter sudden unfavorable environment, protein synthesis will be inhibited, causing the induction or repression of many metabolic pathways according to physiological needs, and the induction of stationary-phase survival genes. This is called "the stringent response". Bacterial alarmone (p)ppGpp functions as a global regulator responsible for the stringent control. Two homologous (p)ppGpp synthetases, RelA and SpoT, have been identified and characterized in *Escherichia coli *[34-37]. Lemos et al. have reported that the *relA *mutation impaired the capacity of *Streptococcus mutans *to form biofilm[38]. No changes in transcription of the relA/spoT homolog(s) were found in 1457*ΔlytSR*. However, SERP1879 encoding an AraC family transcriptional regulator was found to be upregulated significantly in the mutant. Transcriptional regulators of the AraC family are widespread among bacteria and have three main regulatory functions in common: carbon metabolism, stress response, and pathogenesis[39,40].

Among the microarray data, several genes predicted to be involved in anaerobic metabolism were of particular interest. The *arc *operon encodes the enzymes of the arginine deiminase (ADI) pathway, which catalyzes the conversion of arginine into ornithine, ammonia, and CO_2_, with the concomitant production of 1 mol of ATP per mol of arginine consumed. In the absence of oxygen, the ADI pathway enables *S. aureus *to grow in the medium containing arginine [41]. Recent studies demonstrated that the *arc *operon identified in the genome of *S epidermidis *strain ATCC12228 but not in RP62A is located on a novel genomic island termed arginine catabolic mobile element (ACME). Except for the ACME-encoded *arc *operon, all *S. epidermidis *carry a native *arc *operon on the core chromosome. Diep et al. supposed that ACME-encoded gene products might confer survival advantage of *S. aureus *strain USA300 and other ACME-bearing staphylococci within the host, resulting in the widespread dissemination of bacterial progeny [42-44]. In the present study, arginine deiminase activity was performed as previously described [45,46] and 1457*ΔlytSR* exhibited a reduced enzyme activity (Additional file 2, Figure S2).

In the present study, 1457*ΔlytSR* produced slightly more biofilm than its parent strain. However, no genes that are involved in biofilm formation directly, such as *ica *operon encoding enzymes responsible for PIA synthesis, were identified in the transcriptional profile. It was observed that *ica *transcription level and PIA production were similar between 1457*ΔlytSR* and its parent strain. Both tricarboxylic acid cycle stress and anaerobic condition have been proven to induce PIA production and promotion of biofilm, suggesting that changes in the metabolic status can be sensed and regulate biofilm formation [47,48]. Moreover, the stringent response has also been demonstrated to affect biofilm formation[38]. It suggests that *lytSR *mutation may indirectly enhance biofilm formation by altering the metabolic status of *S. epidermidis*.

## Conclusions

The present study suggests that in *S. epidermidis *the LytSR two-component regulatory system play an important role in controlling extracellular murein hydrolase activity and bacterial cell death but has limited effect on autolysis. The *lytSR *mutation invokes a stringent type transcriptional profile, moreover, enhances biofilm formation, which suggests LytSR may function to indirectly regulate biofilm formation by altering the metabolic status of the bacteria, particularly under conditions in which supply of nutrient and oxygen is limited, such as the conditions in biofilm.

## Methods

### Bacterial strains, plasmids and growth media

All the bacterial strains and plasmid used in the present study are listed in Table 3. *E. coli *were cultivated in Luria-Bertani broth (LB), whereas Staphylococcus were grown in B-Medium or Tryptic soy broth (TSB, Oxoid, Basingstoke, England). Unless otherwise stated, all bacterial cultures were incubated at 37 °C, and aerated at 220 rpm with a flask-to-medium ratio of 5:1. SYTO 9 and propidium iodide (PI) (Live_Dead reagents, Molecular Probes, Eugene, OR) were used at a concentration of 1 mM for staining live or dead bacteria in biofilms. Antibiotics were used at the following concentrations: erythromycin, 10 μg ml^-1^, chloramphenicol, 10 μg ml^-1^, ampicillin, 100 μg ml^-1^.

**Table 3:** Bacterial Strains and plasmids used in this study

Strain or plasmid	Relevant characteristic(s)	Source or reference
Strains		
*S. aureus *RN4220	Restriction-negative, intermediate host for plasmid transfer from *E. coli *to *S. epidermidis*	[54]
*S. epidermidis*		
1457	Biofilm-positive laboratory strain	[55]
1457 *ΔlytSR*	*lytSR: : erm *derivative of *S. epidermidis *1457	This study
1457*ΔlytSR *(pNS*-lytSR*)	*lytSR *complementary strain	This study
1457 *ΔlytSR (*pNS*)*	*lytSR *mutant containing the empty cloning vector	This study
1457 *ΔatlE*	*atlE: : erm *derivative of *S. epidermidis *1457	[29]
12228	Biofilm-negative standard strain	[6]
Plasmids		
pBT2	Temperature-sensitive *E. coli-Staphylococcus *shuttle vector. Ap^r ^(*E. coli*) Cm^r ^(*Staphylococcus*)	[49]
pEC1	pBluescript KS^+ ^derivative. Source of *ermB *gene (Em^r^). Ap^r^	[49]
pBT2-*ΔlytSR*	Deletion vector for *lytSR*; *ermB *fragment flanked by fragments upstream and downstream of *lytSR *in pBT2	This study
pNS	*E. coli-Staphylococcus *shuttle cloning vector. Ap^r ^(*E. coli*) Spc^r ^(*Staphylococcus*)	This study
pNS-*lytSR*	Plasmid pNS containing *lytSR *fragment and its native promoter	This study

*Abbreviations: Ap, ampicillin; Cm, chloramphenicol; Em, erythromycin; Spc, spectinomycin

### Construction of the *S. epidermidis lytSR *knockout mutant

In *S. epidermidis *1457 strain inactivation of the *lytSR *operon via homologous recombination using temperature sensitive shuttle vector pBT2 was carried out as described by Bruckner [49]. An XbaI/HindIII-digested erythromycin-resistance cassette (ermB) from plasmid pEC1 was inserted into the pBT2 plasmid, named as pBT2-ermB. The regions flanking *lytSR *operon amplified by PCR were then ligated into the plasmid pBT2-ermB. Primers for PCR were designed according to the genomic sequence of *S. epidermidis *RP62A (GenBank accession number CP000029). Sequences of the primers are listed in Table 4. The homologous recombinant plasmid, designated pBT2-*ΔlytSR*, was first transformed by electroporation into *S. aureus *RN4220 and then into *S. epidermidis *1457. The recombinant strains were grown in B-Medium (10 μg Em ml^-1^) at 30 °C for 16 h, to late-stationary phase. Subsequently, three millilitres of the 30 °C culture was inoculated into 300 ml fresh B-Medium (1:100 dilution) containing 2.5 μg Em ml^-1^. Allele replacement of the temperature-sensitive pBT2-ΔlytSR was achieved following two rounds of growth at 42 °C for 24 h without antibiotic and subsequent selection of Em-resistant (2.5 μg Em ml^-1^) and Cm-sensitive (10 μg Cm ml^-1^) colonies on B-Medium agar plates. Successful replacement of the *lytSR *operon via homologous recombination and loss of the plasmid pBT2-ΔlytSR were verified by PCR and direct sequencing. For analysis of physiological and biochemical changes in the mutant, a GPI-vitek test system was used according to the manufacturer's instructions (BioMerieux Vitek, Hazelwood, Mo, USA).

**Table 4:** Primers used in this study

Primers	Sequence(5'→3')*	Restriction
**Primers used for PCR products in allelic gene replacement**		
*lyt-*UF (upstream fragment)	CCGGAATTCGAACCGATGGACCAGTAG	BamHI
*lyt-*UR (upstream fragment)	CGGAATTCTAAAGAGGGACGACAATGG	EcoRI
*lyt-*DF (downstream fragment)	CCCAAGCTTCAACAACTCGGTCTTCAA	HindIII
*lyt-*DR (downstream fragment))	CTAGCTAGCAAAGGTATGGGAATGACG	NheI
**Primers used in complementation of 1457*ΔytSR1 *strain**		
*lyt-*CF	GGGGTACCTTATTGAAGACCGAGTTGTTGTTTA	BamHI
*lyt-*CR	CGGGATCCTATGAAACAAGCCAATGTAAGTGC	KpnI
**Primers used for real time RT-PCR in confirmation of microarray data**		
gyrB*-*RF	TTTCACTTTCTTCAGGGTTCTTAC	
*gyrB*-RR	CCATCTGTAGGACGCATTATTG	
*lrgA-*RF	GCATTGTGAAATTAGGTCAAGTTG	
*lrgA*-RR	ACTAATAATTGTGACGCAAAGCC	
*serp2169-*RF	GCATCCGCTTCTCCAATATCTG	
*serp2169*-RR	TAAACAACATACACACGCTAAACC	
*ebsB-*RF	TTTGATGCTGCGACTAAAGG	
*ebsB*-RR	CATTGCTGCCCATTCTGC	
*arcA-*RF	GGCTGACTCATACATCTTGG	
*arcA*-RR	GGGTTGTGGTGACATACG	
*leuC-*RF	CCAGGATGTTCTATGTGCTTAGG	
*leuC*-RR	CGCCTTTGCCTTGTCTTCC	

* Primers were designed according to the genomic sequence of *S. epidermidis *RP62A (GenBank accession number CP000029).

### Complementation of 1457*ΔlytSR* with pNS*-lytSR*

For complementation of 1457*ΔlytSR *strain, the staphylococcus cloning vector pCN51 was modified by replacing the erythromycin-resistance cassette with the spectinomycin-resistance cassette, named as pNS [50]. The *lytSR *operon encompassing its promoter and ribosome binding site was amplified by PCR with primers *lyt*-CF and *lyt*-CR. The resulting PCR product was then ligated into BamHI and KpnI sites of the pNS vector. The recombinant plasmid allowed the expression of *lytSR *under the control of its native promoter, named as pNS-*lytSR*. The promoter sequences were predicted by using BDGP Neural Network Promoter Prediction software http://www.fruitfly.org/seq_tools/promoter.html. Meantime, the empty vector pNS was electroporated into 1457*ΔlytSR*as a control.

### Morphology of 1457*ΔlytSR* observed with transmission electron microscopy

Strains of *S. epidermidis *1457, *ΔlytSR* and *ΔatlE* were cultured in TSB medium for 16 hours, and resuspended in 2.5% glutaraldehyde in Dulbecco's phosphate-buffered saline (PBS) overnight. After postfixation in osmium tetroxide, the preparations were dehydrated with increasing alcohol concentrations and embedded in Epon 812. Thin sections were cut using a Leica Ultracut R at a thickness of 70 nm, stained with 1% uranyl acetate-lead acetate and examined with a Philips Tecnai-12 Biotwin transmission electron microscope.

### Triton X-100 induced autolysis

To examine the potential role of *lytSR *in the regulation of autolysis in *Staphylococcus epidermidis*, Triton X-100-induced autolysis of 1457*ΔlytSR* was performed as described by Brunskill & Bayles [10]. Bacterial cells of 50 ml were collected from early exponentially growing cultures (OD_600 _= 0.7) containing 1 M NaCl, and the cells were pelleted by centrifugation. The cells were washed twice with 50 ml of ice-cold water and resuspended in 50 ml of Tris-HCl (pH 7.2) containing 0.05% (vol/vol) Triton X-100. Autolysis was measured during incubation at 37 °C as the decrease in turbidity at 600 nm_, _using a model 6131 Biophotometer (Eppendorf, Hamburg, Germany).

### Zymogram

To determine if the *lytSR *mutation affects murein hydrolase activity, zymographic analysis of extracellular, cell wall-associated murein hydrolases from strains 1457 and 1457*ΔlytSR* grown in TSB medium was carried out essentially as described previously [12,51]. Cell-wall-associated murein hydrolases were extracted with 4% SDS. Briefly bacteria cells from overnight cultures were pelleted down, washed twice with 100 mM phosphate buffer and resuspended by 100 mM sodium phosphate buffer containing 4% SDS in amount about equal to wet weight of pellet. The cell suspension was incubated at 37 °C water bath for 10 min. The supernatant containing surface proteins were collected after centrifugation. Extracellular and cell surface proteins extracted were separated in SDS-polyacrylamide gel electrophoresis gels containing 2.0 mg of *M. luteus *or *S. epidermidis *cells/ml. Murein hydrolase activity was detected by incubation overnight at 37 °C in a buffer containing Triton X-100, followed by staining with methylene blue.

### Cell wall hydrolysis assays

To quantify the amount of hydrolysis observed in the zymographic analysis, cell wall hydrolysis assays were examined as described by Groicher et al. [12]. Extracellular murein hydrolases of bacteria were isolated from 15 ml of a 16-h culture by centrifugation at 6,000 *g *for 15 min at 4 °C. The supernatant was filter-sterilized and concentrated 100-fold using a Amicon Ultra-15 Centrifugal Filter unit (Milipore, 5 kD). The concentration of total proteins in each preparation was determined using the Bradford assay according to the manufacturer's directions. Briefly, 100 μg of enzyme extract was added to a suspension of autoclaved and lyophilized *M. luteus *or *S. epidermidis *cells (1.0 mg/ml) in 100 mM Tris-HCl (pH 8.0) and incubated at 37 °C with shaking. Cell wall hydrolysis was measured as decrease in turbidity at 600 nm every 30 min, using a model 6131 Biophotometer (Ependorf, Hamburg, Germany).

### Detection of Biofilm formation

To investigate the ability of 1457*ΔlytSR* to form biofilm, the standard microtiter-plate test was carried out essentially as described by Christensen et al. [52]. Briefly, overnight cultures of *S. epidermidis *strains grown in TSB medium were diluted 1:200 and inoculated into wells of polystyrene microtiter plates (200 μl per well) and incubated at 37 °C for 24 h. After incubation, the wells were washed gently three times with 200 μl sterile PBS, air-dried and stained with 2% crystal violet for 5 min. Then, the plate was rinsed under running tap water, the crystal violet was redissolved in ethanol and the absorbance was determined at 570 nm.

To determine whether *lytSR *affects cell viability in biofilm, bacterial cells were cultivated in cover-glass cell-culture dish (WPI, Sarasota, FL, USA) as described previously [29]. Briefly, overnight cultures of *S. epidermidis *strains grown in TSB medium were diluted 1:200, then inoculated into the dish (2 ml per dish) and incubated at 37 °C. After 24 hours, the dish was washed gently three times with 1 ml sterile 0.85% NaCl, then stained by SYTO 9 and PI for 15 min and examined by Leica TCS SP5 confocal microscope.

### Quantitative analysis of bacterial cell death inside biofilms

To quantify relative viability of *S. epidermidis *strains, live/dead stained biofilms were scraped from the dish and dispersed thoroughly by pipetting. The integrated intensities (1 second) of the green (SYTO 9, 535 nm) and red (PI, 625 nm) emission of suspensions excited at 485 nm were measured respectively by Beckman Coulter DTX880 multimode detectors. The red/green fluorescence ratios (Ratio^R/G^) were calculated, and a standard curve of Ratio ^R/G ^versus percentage of dead cells in the *S. epidermidis *suspension was plotted as described in the manuals of LIVE/DEAD^® ^BacLight™Bacterial Viability Kit L7012 (Invitrogen, Carlsbad, USA). The percentage of dead cells inside biofilms was determined by comparison to the standard curve.

### Pyruvate utilization test

To verify physiological changes of 1457*ΔlytSR *detected by GPI-vitek test system, overnight cultures of *S. epidermidis *were diluted 1:200 into Pyruvate fermentation broth (Tryptone 10 g, Pyruvate 10 g, Yeast extract 5 g, Dipotassium phosphate 5 g, Sodium chloride 5 g per liter, pH 7.4) and incubated microaerobically at 37 °C [53]. The growth was detected by monitoring turbidity of the cultures at 600 nm.

### RNA extraction and Microarray analysis

Overnight cultures of *S. epidermidis *1457 and 1457*ΔlytSR *were diluted 1:200 into fresh TSB and grown at 37 °C to an OD_600 _of 3.0 (mid-exponential growth). Eight millilitres of bacterial cultures were pelleted, washed with ice-cold saline, and then homogenized using 0.1 mm Ziconia-silica beads in Mini-Beadbeater (Biospec) at a speed of 4800 rpm. The bacterial RNA was isolated using a QIAGEN RNeasy kit according to the standard QIAGEN RNeasy protocol.

The custom-made *S. epidermidis *GeneChips (Shanghai Biochip Co., Ltd) included qualifiers representing open reading frame (ORF) sequences identified in the genomes of the *S. epidermidis *strain RP62A, as well as unique ORFs in *S. epidermidis *strain 12228. The GeneChips were composed of cDNA array containing PCR products of 2316 genes and oligonucleotide array containing 252 genes. Reverse transcription were performed using 2 μg of total RNA using T7 promoter primers and M-MLV reverse transcriptase (Promega, Madison, WI, USA), and then cRNA was transcribed from the resulting cDNA as template. cRNA prepared form 1457*ΔlytSR *and the parent strain was labelled using the dyes Cy3 and Cy5 according to the manufacturer's instructions(Amersham, Piscataway, New Jersey) respectively. Microarray hybridization (at 42 °C for 16 h) and washing of the slides at 50 °C were performed according to the manufacturer's instructions. Hybridized slides were scanned by Agilent Scanner (G2655AA) at a 10-μm resolution. Data of each image were normalized to the mean ratio of means of all features. Mean values and standard deviations of gene expression ratios based on three spot replicates on each microarray were calculated in Microsoft Excel XP. The complete set of microarray data was deposited in the National Center for Biotechnology Information Gene Expression Omnibus (GEO, available at http://www.ncbi.nlm.nih.gov/geo/ and is accessible through GEO Series accession number GSE20652.

### Validation of microarray data by Real time PCR

To confirm the results of the microarray data, the relative expression levels of the *lrgA*, *ebsB*, *arcA*, *serp2169 *and *leuC *genes were determined by real-time PCR with gene-specific primers, designed according to the genomic sequence of *S. epidermidis *RP62A (GenBank accession number CP000029). The sequences of the primers are shown in Table 4. Briefly, DNase-treated RNA was reverse transcribed using M-MLV and a hexamer random primer mix. Appropriate concentration of cDNA sample was then used for real-time PCR using an ABI 7500 real-time PCR detection system, gene-specific primers, and the SYBR Green I mixture (Takara, Dalian, China). Relative expression levels were determined by comparison to the level of *gyrB *expression in the same cDNA preparations.

### Statistical analysis

Experimental data obtained were analyzed with the SPSS software and compared by Student's t test. Differences with P < 0.05 were considered statistically significant.

## Competing interests

The authors declare that they have no competing interests.

## Authors' contributions

TZ performed most of the experimental work and drafted the manuscript. QL carried out real time RT-PCR experiments. JH and FY participated in microarray analysis and corrected the manuscript. DQ and YW directed the project and analyzed data. All authors read and approved the final manuscript.

## Supplementary Material

Additional file 1 **Figure S1**. Validation of *S. epidermidis *1457 *ΔlytSR *strain by PCR analysis. Click here for file

Additional file 2 **Figure S2**. Arginine deiminase activity assays for *S. epidermidis*. Click here for file
