# Supplementary material for: Impact of the Staphylococcus epidermidis LytSR two-component regulatory system on murein hydrolase activity, pyruvate utilization and global transcriptional profile
Source: BMC Microbiol. 2010 Nov 12;10:287. doi: 10.1186/1471-2180-10-287 (PMC2996381; doi:10.1186/1471-2180-10-287)

## Figure S1 - Validation of *S. epidermidis* 1457 *ΔlytSR* strain by PCR analysis

Lane 1: *lytSR* downstream fragment plus *erm* fragment (3.0kb); Lane 2: *lytSR* upstream fragment plus *erm* fragment (2.8kb); M: DL15000bp DNA marker.


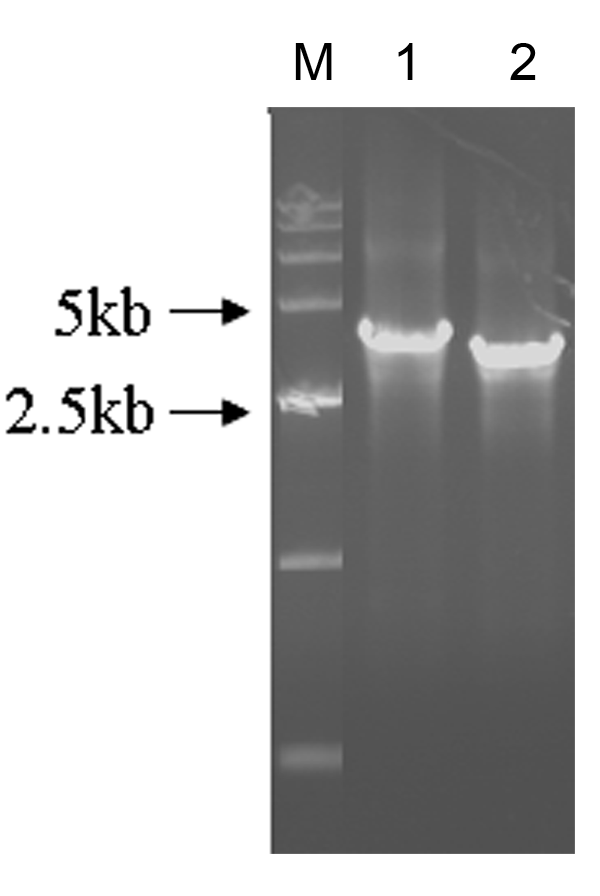

Supplement: Additional file 1 — Figure S1. Validation of S. epidermidis 1457 ΔlytSR strain by PCR analysis. [file 1471-2180-10-287-S1.DOC]
