# Supplementary material for: Impact of the Staphylococcus epidermidis LytSR two-component regulatory system on murein hydrolase activity, pyruvate utilization and global transcriptional profile
Source: BMC Microbiol. 2010 Nov 12;10:287. doi: 10.1186/1471-2180-10-287 (PMC2996381; doi:10.1186/1471-2180-10-287)

## Figure S2 - Arginine deiminase activity assays for *S. epidermidis*

Bacteria were grown statically for 18 hours in TSB supplemented with 20 mM arginine. Cell-free lysates of *S. epidermidis* were prepared as follows. Aliquots (5 ml) were harvested by centrifugation, washed with cold 0.01 M potassium phosphate buffer (pH 7.0), and then homogenized using 0.1mm Ziconia-silica beads in Mini-Beadbeater. Arginine deiminase activity assays were performed essentially as described previously [39]. In brief, cell-free lysates (100 μl) were added to Eppendorf tubes containing prewarmed (37 °C) reaction buffer (400 μl) (5.8 mM L-arginine and 131 mM potassium phosphate [pH 5.8]) and incubated at 37 °C for 15 min. After 15 min, the reaction was stopped by the addition of 50 μl of ice-cold 70% perchloric acid and the samples were clarified by centrifugation. The supernatant (200 μl) was analyzed for citrulline production as described. One unit of arginine deiminase activity was defined as the amount of enzyme that catalyzed the formation of 1 μmol of citrulline. Data are means ± SEM of 3 independent experiments. ^＊^P ＜ 0.05; *ΔlytSR* vs. WT; *ΔlytSR(*pNS*-lytSR)* vs. *ΔlytSR(*pNS*-lytSR)*.


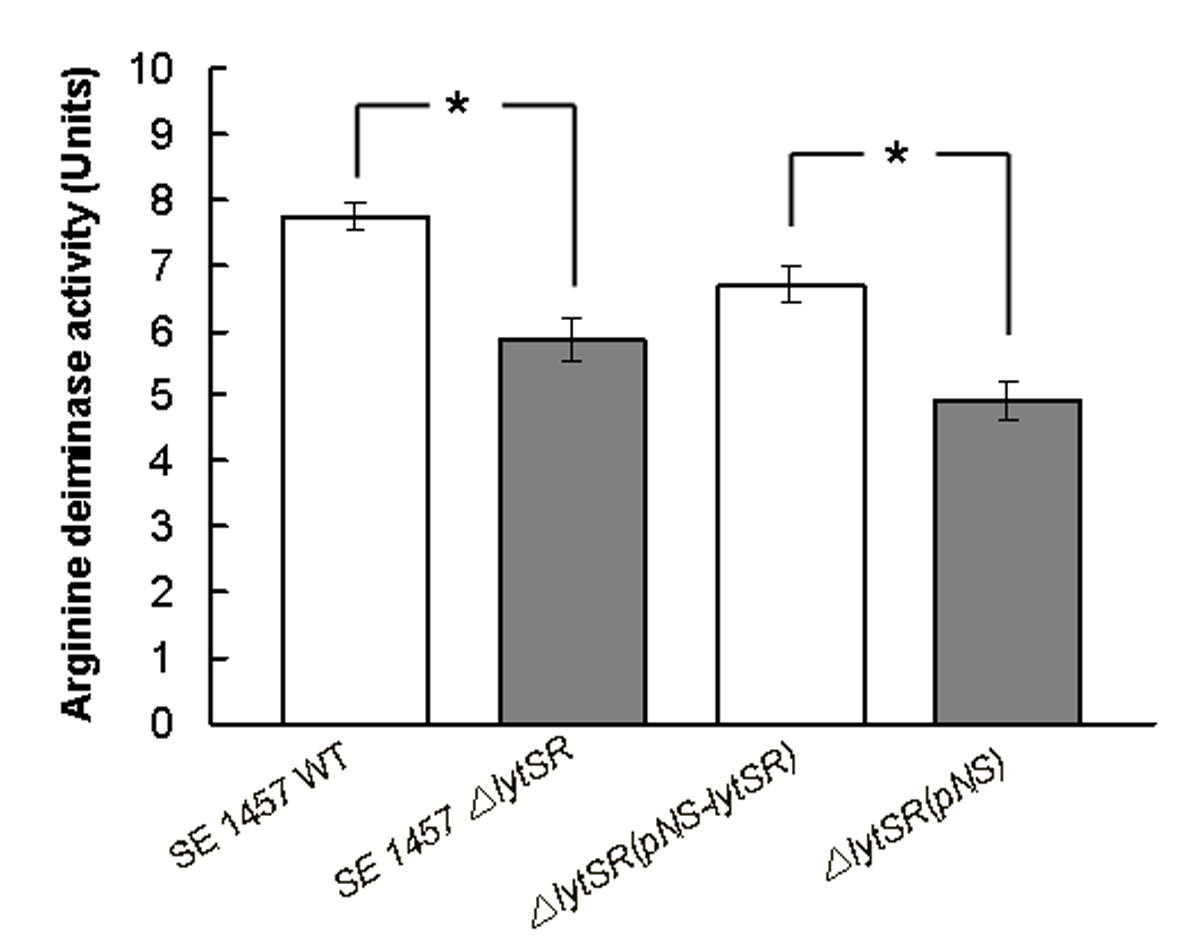

Supplement: Additional file 2 — Figure S2. Arginine deiminase activity assays for S. epidermidis. [file 1471-2180-10-287-S2.DOC]
